# Explainable Cluster-Based Predictive Framework for Early Diagnosis of Autism Spectrum Disorder Using Behavioral Biomarkers

**DOI:** 10.3390/diagnostics15243241

**Published:** 2025-12-18

**Authors:** Menwa Alshammeri, Zulfiqar Ahmad, Mamoona Humayun, Malak Alamri

**Affiliations:** 1 Department of Computer Science, College of Computer and Information Sciences, Jouf University, Sakaka 72388, Saudi Arabia; 2 King Salman Center for Disability Research, Riyadh 11614, Saudi Arabia; 3 Department of Computer Science and Information Technology, Hazara University, Mansehra 21300, Pakistan; 4 School of Computing, Engineering and the Built Environment, University of Roehampton, London SW15 5PJ, UK; mamoona.humayun@roehampton.ac.uk

**Keywords:** Autism Spectrum Disorder, neuropsychiatric diagnosis, behavioral biomarkers, machine learning, explainable AI, clustering, early screening

## Abstract

**Background/Objectives:** Autism Spectrum Disorder (ASD) is a multifaceted neuropsychiatric condition characterized by early behavioral irregularities that often precede formal diagnosis. Timely and precise detection remains a major clinical challenge due to the complexity of behavioral manifestations and the limited accessibility of diagnostic resources. **Methods:** In this study, we present an explainable machine learning framework for the early diagnosis of ASD using behavioral biomarkers derived from toddler screening data. The framework integrates unsupervised learning (DBSCAN and K-means clustering) to identify latent behavioral patterns, followed by predictive modeling using logistic regression (LR), random forest (RF), and support vector machine (SVM). To ensure transparency and clinical interpretability, a SHAP (SHapley Additive exPlanations) analysis is employed to quantify the contribution of each behavioral feature to the model’s predictions. **Results:** Experimental evaluations reveal that the RF model achieves the highest accuracy (98.85%), followed by SVM (97.70%) and LR (90.53%). The explainability results highlight meaningful and clinically relevant behavioral indicators associated with ASD risk. **Conclusions:** The proposed framework not only enhances diagnostic accuracy but also promotes interpretable AI for real-world integration into neuropsychiatric assessment pipelines.

## 1. Introduction

Autism Spectrum Disorder (ASD) is a lifelong neurodevelopmental condition that affects a person’s ability to communicate, interact socially, and process information [1]. The symptoms and severity of the disease are wide-ranging, and early diagnosis and intervention are difficult. The WHO (2021) estimated that around 1 out of 100 children worldwide is diagnosed with ASD, highlighting the public health significance of ASD [2,3]. According to the Centers for Disease Control and Prevention (CDC, 2022), in regions with advanced screening systems such as the U.S., the prevalence is 1 in 31 children, which has been continuously rising over the last two decades [4]. This is due to increased awareness, better definition of diagnostic criteria, and broader definition of the spectrum. Although much progress has been made in understanding ASD, too many children are not diagnosed until after age four, when intervention has the greatest impact. Data from these statistics emphasize the need to find data-driven, innovative methods for early and accurate detection of autism in different populations [5].

Early detection of ASD is essential in enhancing the opportunity for obtaining effective and favorable treatment outcomes. Early diagnosis has long been shown through research to both improve cognitive, social, and communication skills and support the timely implementation of behavioral and educational interventions [6]. However, conventional diagnostic techniques often depend on clinical attention and subjective identification, which can often be delayed, and involved people may miss subtle behavioral cues, especially in under-resourced or rural settings. To fill in these gaps, one line of thought around ASD screening is emerging in integrating advanced technologies like clustering, predictive modeling, and explainable artificial intelligence (XAI) [7,8,9,10]. Unsupervised clustering of behavioral data allows for unsupervised grouping of data that identify hidden patterns indicating early signs of ASD prior to clinical detection [11]. This allows predictive modeling to use machine learning algorithms to identify individuals who may have ASD, based on behavioral features, with good accuracy, and scale with large amounts of data [12,13,14]. Most importantly, XAI methods guarantee that the decision-making process of these models is interpretable and transparent, which is crucial in the medical context due to trust [15,16,17,18]. By employing these cutting-edge tools together, researchers and clinicians can bring us one step closer to developing a less subjective, faster, and fairer early-detection framework for autism in order to help children and families affected by ASD [1].

The motivation of this study comes from the need to bridge the gap between early behavioral manifestations of ASD and early, accurate diagnosis. Although there is increasing knowledge about the problem, many children are still diagnosed after multiple developmental windows of opportunity, which limits the effectiveness of early intervention strategies. Diagnostic methods used traditionally are time-consuming, resource-intensive, and very often inaccessible in most parts of the world. This points to the need for data-driven, scalable approaches that can provide some support to clinicians and caregivers to pick up early signs of ASD. Today, the advent of artificial intelligence, especially clustering, predictive modeling, and explainable AI, presents strong tools to analyze complex behavioral datasets and to seek hidden patterns that may escape human observation. This study intends to develop an intelligent, interpretable, and automated framework with these technologies to enhance the early detection capabilities. An approach of this kind not only promises improved diagnostic accuracy but also allows for greater transparency and trust in the decision-making process and hence the feasibility of integration in the practice of real-world screening. The main contributions of this article are highlighted below:

• We propose a holistic framework to tackle the issue of early detection of ASD in combination with clustering, prediction modeling, and XAI. The framework is built to extract hidden behavioral patterns in order to support early diagnosis using interpretable machine learning models.
• We use K-means and DBSCAN to cluster behavioral data and find natural groupings; then, we evaluate these and determine which method provides a good representation of ASD characteristics.
• For the predictive analysis, we used logistic regression (LR), random forest (RF), and support vector machine (SVM) and measured the performance of each using accuracy, precision, recall, F1 score, and Receiver Operating Characteristic (ROC) curve.
• We incorporated an XAI technique SHAP (SHapley Additive exPlanations) to visualize the feature importance and provide an actionable approach for understanding model behavior for increased transparency and to aid the interpretability of the predictions.

The rest of the paper is organized as follows: Section 2 provides a discussion of related works. In Section 3, the system design and model are described. In Section 4, the performance evaluation is discussed. In the last section, Section 5, we conclude the article with some future directions.

## 2. Related Work

We reviewed the related work in respect of early detection of ASD, clustering applications, machine learning implementations, and use of explainable AI.

A review article of early detection strategies for Autism Spectrum Disorder (ASD) in primary care and community settings in the United States was presented in [6]. The study stressed that, although precise diagnoses of autism can be made by 24 months of age, many children are not diagnosed until later in childhood, missing important early intervention opportunities. The authors reviewed 40 studies from 1990 to 2013 that were grouped into awareness initiatives, routine screenings, and practice improvement strategies. While these and other approaches increased the rate of screening and referral, the review noted a large gap: very few studies had measured whether early screening actually decreased the age at which a person was diagnosed or increased the probability that a person was enrolled in services.

The framework presented in [7] is a novel two-phase framework for the early identification of ASD and the personalization of educational strategies for affected children. Sequences of behaviors among toddlers were merged using two ASD screening datasets, and three feature-engineering approaches were used to extract the most important feature of the machine learning models with the purpose of increasing the classification accuracy. In the first phase, ASD was classified using a hybrid ensemble of logistic regression (LR) and SVM with chi-square-based feature selection, and the accuracy was 94. In the second phase, the framework focused on educational personalization by the evaluation of behavioral, verbal, and physical response patterns for the recommendation of the tailored teaching strategies. Matching the most appropriate educational approach to individual ASD profiles was achieved with this component to yield a very impressive 99.29% accuracy.

In [12], the authors had proposed a comprehensive framework to evaluate the performance of different ML classifiers with associated feature scaling techniques for early ASD detection. Four standard ASD datasets (toddlers, adolescents, children, and adults) were used to preprocess with four widely used feature scaling methods: Quantile Transformer (QT), Power Transformer (PT), Normalizer, and Max Abs Scaler (MAS). Finally, eight classic ML classifiers were used to evaluate the processed datasets: RF, LR, SVM, K-Nearest Neighbors (KNN), AdaBoost (AB), Decision Tree (DT), Gaussian Naïve Bayes (GNB), and Linear Discriminant Analysis (LDA). The highest accuracy values of 99.25% for toddlers, 97.95% for children, 97.12% for adolescents, and 99.03% for adults were obtained in the experimental results using AdaBoost and LDA, respectively.

The authors of [19] stressed the urgent need for ASD diagnosis at its early stages, especially since ASD has a lifelong impact on affected individuals and their families. ASD now affects approximately 1 in 66 Canadian children aged 5 to 17, and as such the likelihood of primary care providers (i.e., pediatricians and family physicians) seeing cases of ASD is increasing. The study encourages early monitoring of developmental milestones and suggests evidence-based tools and guidelines that can help healthcare professionals to detect early symptoms of ASD. The main argument is that early diagnosis allows for early access to behavioral and educational interventions, the most effective interventions at times when the neuroplasticity of the developing child is at its highest. The motivation for the presented framework motivation is meant to support early detection through automated, data-driven methods that work in conjunction with clinical judgment, facilitate early intervention planning, and lead to earlier diagnosis.

The authors of [20] explored the ability of machine learning for early detection of ASD via both classification and clustering approaches. When they applied eight state-of-the-art classification models on multiple ASD datasets, including children, adults, and combined data, they found that near-perfect accuracies were achieved with SVM and LR, achieving 100% (for children) and 97.14% (for adults). Furthermore, when their Artificial Neural Network (ANN) model was finely tuned, it obtained an accuracy of 94.24% on the combined dataset. The study also evaluated unsupervised clustering methods where true labels do not exist beyond supervised learning. It was assessed based on metrics like Normalized Mutual Info (NMI), Adjusted Rand Index (ARI), and Silhouette Coefficient (SC), and spectral clustering.

Recent studies have reemphasized the need for early detection and early intervention for ASD, where early diagnosis was shown to significantly augment long-term developmental outcomes. While traditional approaches to foster awareness, routine screening, and improvement strategies in community and clinical settings have been initiated, many of them do not include measurable outcomes related to reducing diagnosis delays or improving access to services. However, machine learning models used for the identification of ASD based on classification with various feature engineering methods have shown high accuracy; yet, they are not suitable for clinical adoption due to their being black boxes with limited transparency. Moreover, though some research combines behavioral data plus education strategies, no effort has been made to combine unsupervised learning for pattern discovery with explainable AI techniques for trust and understanding. To close these gaps, the proposed framework combines clustering algorithms for identifying hidden patterns with predictive modeling for accurate classification, as well as SHAP-based explainability for interpretable insights; thus, the transparency, accuracy, and practicality of early ASD detection is improved.

## 3. System Design and Model

**Figure 1:**
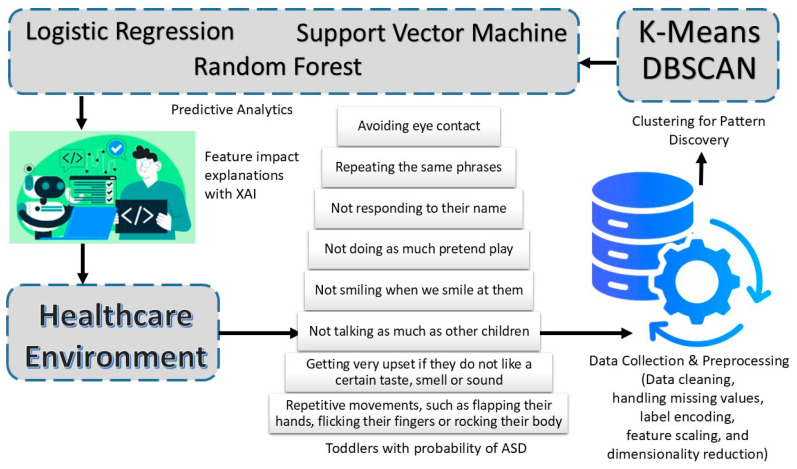
Early detection of ASD by integrating clustering, predictive modeling, and XAI.

In this study, we present a holistic framework for early ASD detection incorporating clustering, predictive modeling, and XAI techniques. Figure 1 shows an illustration of the overall system design. The primary source of analysis is obtained from behavioral data through multiple preprocessing steps such as data cleaning, handling missing values, label encoding, feature scaling, and dimensionality reduction to provide quality input to downstream tasks. Latent patterns and subgroups hidden in the data are uncovered using the clustering component; these are not usually revealed through traditional labeling. This unsupervised step helps in identifying the natural groupings of behavioral traits that may be early signs of ASD. Principal Component Analysis (PCA) was applied to reduce the high-dimensional behavioral dataset into lower dimensions while preserving the majority of variance. This step improves computational efficiency and interpretability before clustering. We implemented two techniques for clustering, i.e., K-means and DBSCAN. K-means was chosen for its efficiency and suitability in identifying well-separated spherical clusters, while DBSCAN was implemented due to its ability to detect clusters of arbitrary shapes and to handle noise efficiently. The combination of both methods allows a balanced comparison between centroid-based and density-based clustering approaches to ensure reliable identification of behavioral patterns in the ASD dataset.

In the predictive modeling phase, individuals were classified based on their behavioral characteristics, and the probability of their having ASD was assessed. Finally, we applied supervised learning algorithms, i.e., LR, RF, and SVM. To determine the best approach, these models were evaluated using the metrics of accuracy, precision, recall, F1 score, and ROC curve. We used SHAP (SHapley Additive exPlanations) as an XAI technique to explain the contribution of each feature to the model’s output, thus clarifying how the model makes decisions. The use of this integrative approach ensures simultaneous high performance, trustworthiness, and explainability in early ASD detection, which are key factors for the clinical adoption of a model in real life.

- Algorithm for the proposed framework

Algorithm 1 for the proposed framework incorporates clustering, predictive modeling, and explainable AI, which aids in the early detection of ASD from behavioral data. The raw dataset initially undergoes preprocessing steps such as missing value imputation, label encoding, and min–max normalization to ensure that the data quality and numbers were suitable for the modeling process. In the first phase, clustering was used to find intrinsic patterns in the data. K-means and DBSCAN were implemented and compared. The K-means process groups instances, such that the intracluster variance is minimized; meanwhile, DBSCAN clustering is performed based on density and thus is capable of dealing with noise and arbitrary-shaped data distributions. The preprocessed clustered data are used in the predictive modeling phase, in which several supervised machine learning models are trained, including LR, RF, and SVM. These models predict whether a child is showing signs of ASD, and their performance is evaluated using standard classification metrics, including accuracy, precision, recall, and F1 score. The final phase of the framework addresses explainable AI with SHAP for the purpose of enhancing transparency and supporting clinical decision making. SHAP quantifies the contribution of each feature to the model’s prediction and gives an interpretation of the model’s decision making. On the one hand, this integrated approach guarantees high accuracy and robustness in ASD detection; on the other hand, it enhances the interpretability and trustworthiness issue in ASD diagnosis aided by AI.
**Algorithm 1:** Integrated Framework for Early ASD Detection Using Clustering, Predictive Modeling, and Explainable AI**Input:**  Behavioral dataset
D={x1,x2,…,xn} with features
$x_{i}\in R^{m}$       Labels
Y={y1,y2,…,yn}, where
$y_{i}\in \{0,1\}$ (0 = No ASD, 1 = ASD) **Output:**  Predicted labels
$\hat{Y}$. SHAP-based explanations for model predictions **1.** **Preprocessing:**•Missing value imputation
$$x_{ij}=\;\left\{\begin{array}{c} x_{ij},\;if\;x_{ij}\neq null\;\\ \mu_{j},\;otherwise\; \end{array}\;where\;\mu_{j}=\;\frac{1}{n}\sum_{i=1}^{n}x_{ij}\;\right.$$•Label Encoding/One-Hot Encoding for categorical variables•Apply min–max normalization:
$${x^{\prime }}_{ij}=\;\frac{x_{ij}-\min\left(x_{j}\right)}{\max\left(x_{j}\right)-\min\left(x_{j}\right)}$$**2.** **Clustering for Pattern Discovery:**•Let $X\in R^{n\times m}$ be the preprocessed feature matrix•DBSCAN: Parameters = {ϵ, MinPts}, For each point $x_{i}$, define neighborhood
$$N_{\epsilon }\left(x_{i}\right)=\{x_{j}\;|\;∥\;x_{i}-x_{j}∥\;\leq \;\epsilon \}$$  A point is a core point if $|N_{\epsilon }\left(x_{i}\right)|\;\geq \;MinPts$•K-Means Clustering: Initialize k centroids {μ1,…,μk}•Minimize:
argminc ∑i=1k∑xϵCi∥ x− μi∥2**3.** **Predictive Modeling:**•Train supervised models using labeled data (*X*, *Y*)•For each model M ϵ {LR, RF, SVM}, fit
$M\;:X\to \;\hat{Y}$•Compute accuracy (*A*), precision (*P*), recall (*R*), and *F*1 score
$$A=\;\frac{TP+TN}{TP+TN+FP+FN\;}$$
$$P=\;\frac{TP}{TP+FP\;}$$
$$R=\;\frac{TP}{TP+FN\;}$$
$$F1-Score=\;\frac{2(P\times R)}{P+R\;}$$**4.** **Explainable AI with SHAP**•Compute SHAP values $\phi_{j}$ for each feature j for instance $x_{i}$:
$$f\left(x_{i}\right)=\phi_{0}+\;\sum_{j=1}^{m}\phi_{j}$$•Rank features by their SHAP contributions ${|\phi }_{0}|$ to interpret predictions.**5.** **Output**•Return:Predicted label ${\hat{y}}_{i}=M\left(x_{i}\right)$Feature impact explanations $\left\{\phi_{1},\;\phi_{2},\ldots ,\;\phi_{m}\right\}$

b.  Dataset

We have utilized a dataset called “Autism screening data for toddlers” [21], which is publicly available on the Kaggle platform. The dataset was developed to assist in the research of early detection of ASD in young children. The data for this dataset was collected through the ASDTests mobile application. The app was developed to support the first step to ASD screening of toddlers by using a structured questionnaire. The collection of the data followed the ethical guidelines and received formal approval for the use of personal health-related information. The dataset has several hundred instances, each instance being a single toddler. Each record contains a set of demographic and behavioral features for the evaluation of traits that may be ASD-related. The demographic attributes include the toddler’s age (in months), gender, ethnicity, and family history of autism. Ten binary or categorical questions of the behavioral screening section pertain to typical early symptoms of ASD, such as problems in communication, lack of eye contact, lack of interest in social interaction, or repetitive behaviors. Each of these behavioral features is meant to be a sign of an early indicator of autism. The final class label is a key component of the dataset, and it indicates whether a child is deemed to exhibit signs of ASD in the screening. In the existing labeled data, this binary classification (ASD or no ASD) is compatible for researchers to train and evaluate machine learning models for a predictive analysis. And the dataset includes information about place of residence and whether the screening was performed by a parent or guardian, which might make it possible to better understand the responses. Because this dataset is particularly useful for building models aiming at early ASD detection with data-driven approaches, it is worth using it in the following way. This allows experimentation with supervised learning techniques, clustering methods, and explainable AI models to improve diagnostic accuracy and interpretability.

## 4. Performance Evaluation

We perform simulations and evaluate the performance of proposed framework regarding early detection of ASD.

- Evaluation Metrics

We evaluated the performance of the models implemented in the proposed framework using silhouette score, accuracy, precision, recall, F1 score, and ROC curve. We calculated accuracy, precision, recall, and F1 score based on the following terms:

• True positives (TP): The number of correctly identified positive instances.
• True negatives (TN): The number of correctly identified negative instances.
• False positives (FP): The number of incorrectly identified positive instances.
• False negatives (FN): The number of incorrectly identified negative instances.

b.  Experimental Design

The experiments were performed by implementing two clustering methods, K-means and DBSCAN and three ML models, random forest, logistic regression, and support vector machine [22,23,24,25,26]. The dataset has been divided into two parts: the training set and the test set. The training set comprised 80% of the total records in the dataset. The test set comprised 20% of the total number of records. All experiments were conducted using Python (version 3.10.12) in a GPU-based environment. Predefined machine learning packages and libraries were utilized, including Pandas (2.2.2), NumPy (1.26.4), Matplotlib (3.9.2), Seaborn (0.13.2), and Scikit-learn (1.5.2), which provided LabelEncoder and OneHotEncoder functionalities for preprocessing.

c.  Dataset Preprocessing

Before applying clustering and predictive modeling, preprocessing steps were performed. The dataset was examined for typographical inconsistencies, and column names were standardized (for instance, the attribute “Jauundice” was corrected to “Jaundice”). The dataset exhibited minimal missing values, less than 2%, primarily in demographic features such as age and ethnicity, which were imputed using mean and mode imputation for numerical and categorical attributes, respectively. Mean imputation was selected due to the low proportion of missing data (<2%) and the non-complex structure of the dataset. To prepare the data for analysis, categorical features were encoded using the LabelEncoder function from scikit-learn. Binary categorical attributes, including Sex, Jaundice, Family_ASD, and Class, were transformed into numerical form to make them compatible with clustering and classification algorithms. After encoding, the target label (class), indicating ASD or non-ASD, was separated from the feature set for unsupervised learning. The remaining feature space was standardized using the StandardScaler method to normalize all attributes to a common scale.

- Results and Discussion

- Clustering for Pattern Discovery

**Figure 2:**
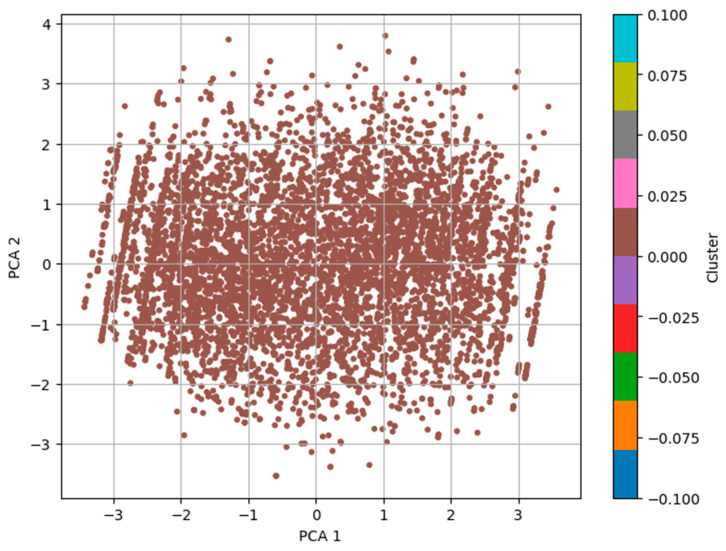
DBSCAN clusters with PCA.

We started our analysis by applying the DBSCAN (Density-Based Spatial Clustering of Applications with Noise) algorithm. It was chosen because DBSCAN has a strong performance in detecting the clusters of different shapes and is robust to noise. We applied DBSCAN directly to the scaled feature space. The silhouette score was 0.6068, which is a moderate–strong clustering structure, with 220 clusters, excluding noise points. We further improved cluster quality and supported the visualization by reducing the feature space to three principal components using PCA [27]. The data were then reduced using PCA, and DBSCAN was reapplied to this data using an eps value of 7.0. Figure 2 shows the clustering outcome, where the cluster formation is based on the first two principal components. First, using raw features and then PCA-reduced data to create this two-step approach, we are able to compare the clustering performance and the interpretability of the model while reinforcing the model’s ability to detect the inherent groupings in the behavioral dataset.

**Figure 3:**
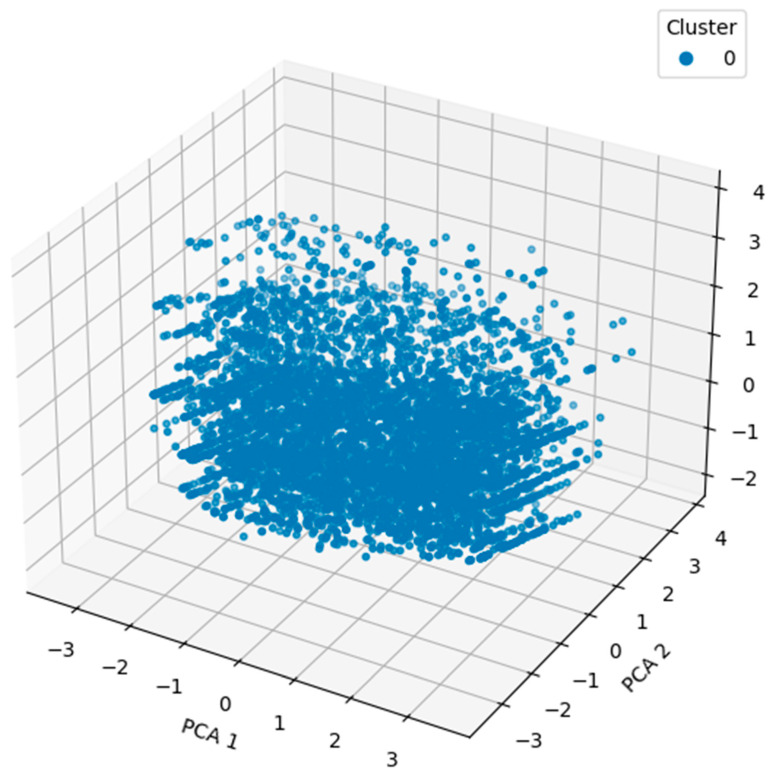
Three-dimensional scatter plot of the clustering result obtained using DBSCAN on the PCA-reduced data.

Figure 3 shows a three-dimensional scatter plot of the clustering result obtained using DBSCAN on the PCA-reduced data. We apply the DBSCAN algorithm to the reduced original high-dimensional behavioral dataset, with PCA reducing the original three dimensions to three principal components. The cluster labels are assigned to each point in the 3D space, which corresponds to that of the toddler’s behavioral profile projected onto the top three principal components. The visualization in this case gives a more complete picture of how the cluster separation in the reduced feature space appears, with obvious groupings and the spatial relations between the clusters. This implies that the PCA transformation retained important structural information contained in the data and is useful in applying DBSCAN to identify underlying patterns in the data. Interpretability is improved using 3D visualization, as depth and distribution variations cannot be seen in 2D plots.

DBSCAN performed well with a high silhouette score of 0.6068, but the large number of clusters (220) produced by the full dataset was difficult to interpret. The loadings were examined to make them easier to interpret, and PCA was used to reduce dimensionality to three components. Hence, it turned out that, in this reduced space, DBSCAN yielded much clearer information, namely three distinct clusters with a silhouette score of 0.2388. The clusters formed will generally correspond to true subpopulations in the set and will provide a useful basis for further analysis. Two- and three-dimensional visualizations support the observation of a central cluster and some weaker peripheral subgroups, possibly associated with behavioral or demographic differences. Thus, we implemented K-means clustering as a complementary method of understanding underlying patterns in the behavioral dataset for ASD screening. The partition-based clustering algorithm, K-means, is widely used to divide the dataset into a preassigned number of clusters by minimizing intracluster variance. Compared to the DBSCAN algorithm, the density-based approach assumes the cluster shapes are spherical and equal in size. This was performed so as to compare various strategies of clustering in the data and determine which is better in terms of revealing structure in the same. For our analysis, we assume that the dataset may naturally separate into groups of toddlers with and without ASD tendencies and set the number of clusters k = 2. Cluster 1 represents toddlers with higher scores in behavioral indicators such as limited social interaction and reduced eye contact, which are typically associated with a higher likelihood of ASD. In contrast, cluster 2 consists of toddlers with comparatively lower behavioral risk scores, indicating milder or no ASD tendencies. While the dataset does not explicitly categorize ASD subtypes, the clustering patterns align behavioral severity gradients. The K-means algorithm was applied to the scaled dataset after ensuring robust cluster centroids through multiple initializations. The silhouette score was used to evaluate the clustering outcome, and it gave a value of 0.1536, which showed weaker cohesion and separation between clusters than that of DBSCAN.

**Figure 4:**
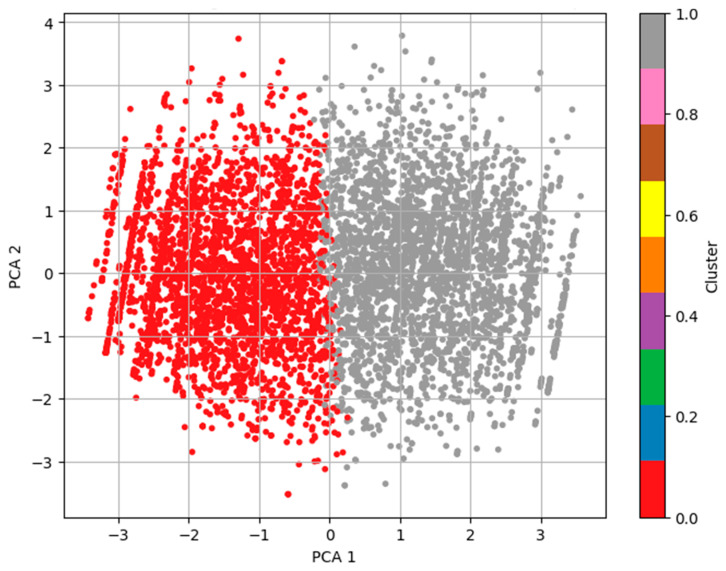
K-means clustering with k = 2 visualized in PCA-reduced 2D space.

To visualize the clustering result, we performed a PCA to reduce the data into two dimensions. Figure 4 shows the resulting scatter plot for a subject, and the color in the plot denotes the cluster to which the subject is assigned. The clusters are somewhat distinguishable in the plot, and the low silhouette score hints at the fact that K-means was not the best method to capture the nonspherical and overlapping distribution in the behavioral data compared to DBSCAN.

In this way, we compare K-means (k = 2) and DBSCAN via PCA (3D) as unsupervised clustering techniques to identify hidden patterns from autism screening data. After applying this technique under the PCA reduced space, the K-means centroid-based algorithm has a very clear linear separation with a silhouette score of 0.1536. In comparison to the case discussed above, DBSCAN adds along with 3D PCA, which has at once given a lower silhouette metric score of 0.2388, and without any predefining of cluster number, naturally cluster 3 meaningful clusters identified. The results of this density-based technique were better for treating and detecting the outliers in a more realistic way, as can be seen from their distance visualization in 2D and 3D. The clustering in this case also produced the very same results as naturally distributed data and also found its subgroups that could not be found with the help of K-means under the same conditions. In this case, DBSCAN with PCA was best, as it outperformed the others, allowed more flexibility in nonlinear structures, and had far more interpretability in the real-life situation of autism trait screening.

ii.  Integration of Clustering Labels into Predictive Modeling

In order to increase the effectiveness of predictive analysis, clustering results were used as an additional feature in the supervised learning phase. The goal of this approach is to utilize unsupervised insights unearthed as part of clustering (i.e., hidden patterns and natural groupings in the data) to augment the input space of the predictive models. Intuitively, if these cluster labels contain latent structural information about ASD behavioral traits, then they can help in improving the classification accuracy. We implemented and tested three machine learning models, i.e., logistic regression (LR), random forest (RF), and support vector machine (SVM). LR is well-suited baseline linear model for binary classification, and RF is an ensemble-based method that is robust and accurate. SVM is a powerful model especially good in high-dimensional space. The behavioral dataset was augmented with the clustering labels obtained from the best-performing unsupervised technique in order to train each model. Therefore, we combined the outputs of clustering with supervised learning in order to exploit the inherent structure of the data as well as label-driven relationships to improve the overall performance and interpretability of the ASD prediction. Table 1 provides a summary of the key hyperparameters along with description for the models implemented in the proposed framework. These parameters were selected based on standard configurations recommended in the scikit-learn library.

In Table 2, a detailed performance comparison is presented. We evaluate using four metrics: precision, recall, F1 score, and accuracy separately for both classes (non-AS and ASD) and then calculate macro and weighted averages. All three models performed well for the ASD class (0). The random forest model outperformed the other models, having accuracy of 0.9885, and SVM also had a very good accuracy 0.9770. More importantly, RF and SVM were much better than LR for the ASD class (1), since this is an important class in early detection. With 0.99 precision, 0.98 recall, and a 0.98 F1 score, random forest proved that it is capable of correctly identifying ASD cases without too many false negatives or false positives. Logistic regression was less precise, with 0.84 precision, 0.85 recall, and a 0.84 F1 score, due to its linear shortcomings for handling more complicated patterns in the data. The best model is random forest, which performs almost perfectly in classifying ASD and non-ASD classes. Although logistic regression is more interpretable, it might not be enough for high-stakes ASD detection, as it has lower recall for ASD cases.

**Figure 5:**
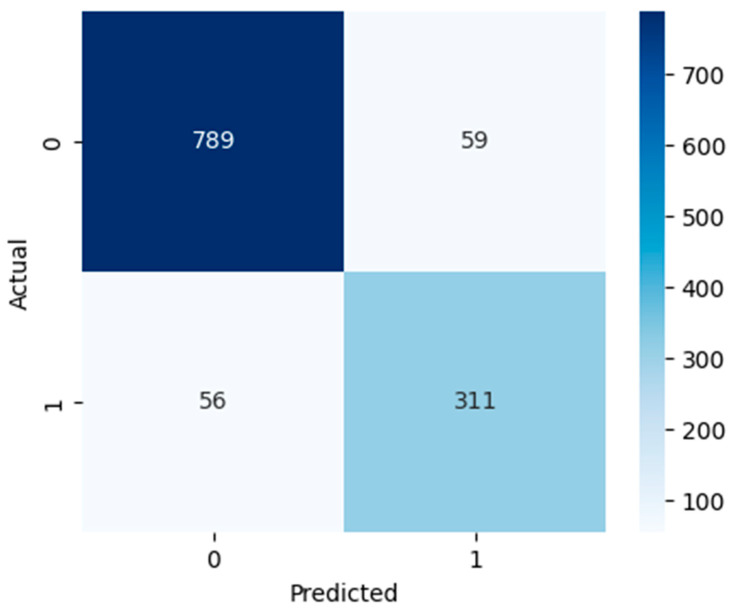
LR confusion matrix.

**Figure 6:**
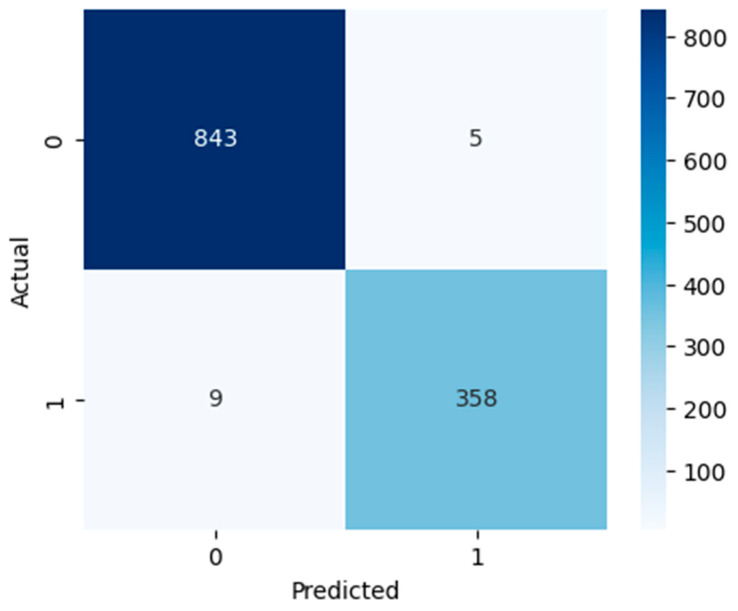
RF confusion matrix.

**Figure 7:**
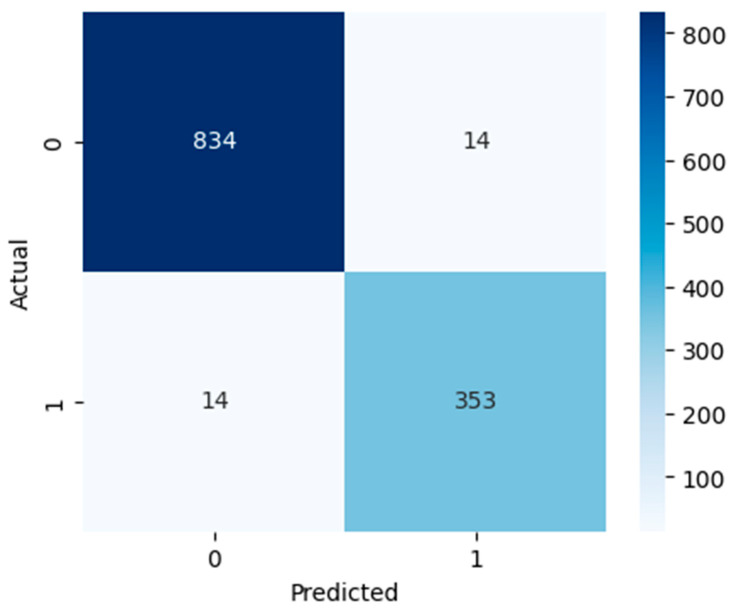
SVM confusion matrix.

Figure 5, Figure 6 and Figure 7 show confusion matrices that show deeper insight into the classification performance. The models are interpreted beyond overall accuracy, with each matrix representing the number of true positives (TP), false positives (FP), true negatives (TN), and false negatives (FN) for each matrix. For the model, TN and TP were 789 non-ASD cases and 311 ASD cases, respectively. Nevertheless, it misclassified 59 ASD cases as non-ASD (FP) and 56 non-ASD cases as ASD (FN). The relatively larger number of false positives and false negatives compared to the other models suggests that, while logistic regression has an overall accuracy of 90.53%, which is not bad, it is less precise and may not perform well in a real-world ASD screening scenario if a minimal misclassification rate is required. However, as indicated by the RF model, it has high classification power, recognizing 843 non-ASD (TN) and 358 ASD cases (TP), and has only five false positives and nine false negatives. This yields a very high accuracy overall of 98.85%; this is a high accuracy for such a model that has relatively low error rates. In the context of early and accurate detection, RF is a strong candidate for practical deployment in clinical, pre-screening, or other applications due to the near-perfect separation of ASD and non-ASD instances. The SVM model also does well, predicting 834 out of 1187 non-ASD (TN) and 353 out of 445 ASD cases (TP), with only 14 non-ASD and 14 ASD misclassifications. This results in 97.70% accuracy, and although this is slightly lower than RF, it is still quite reliable. It is found that SVM’s balanced error distribution also indicates that it can learn more complex boundaries between ASD and non-ASD features, with slightly higher misclassification than RF.

**Figure 8:**
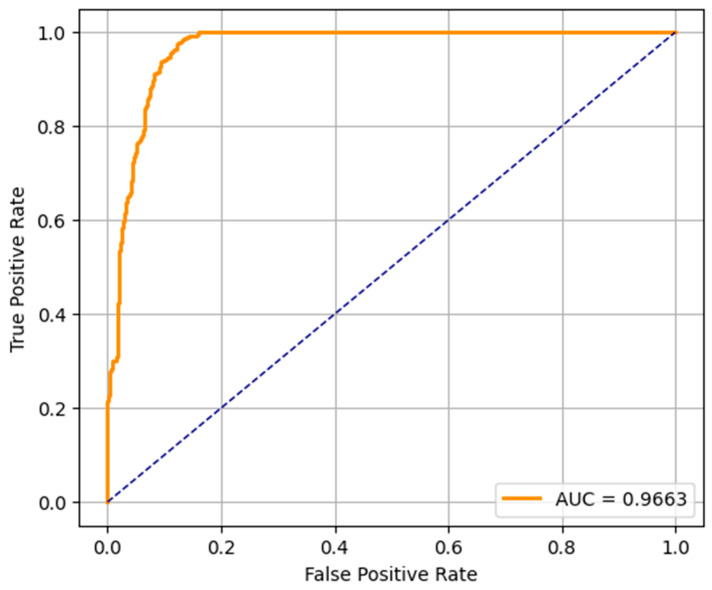
LR-ROC curve.

**Figure 9:**
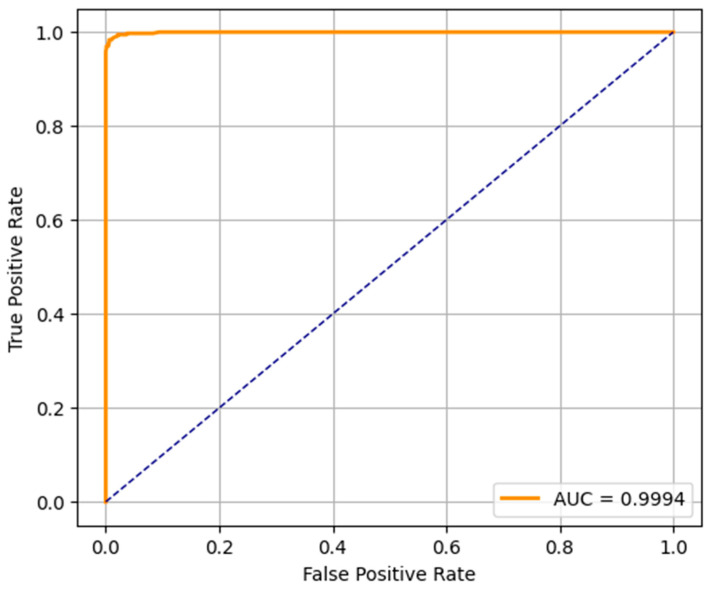
RF-ROC curve.

**Figure 10:**
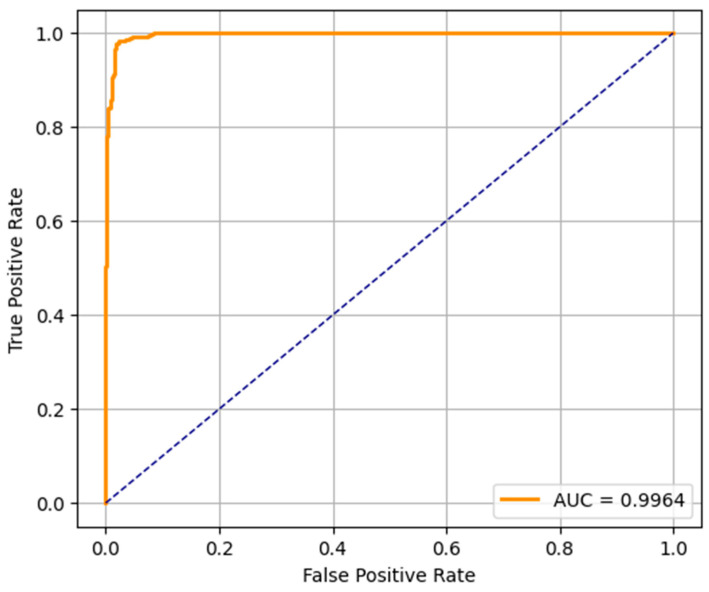
SVM-ROC curve.

The ROC curves of the three predictive models (LR, RF, and SVM) applied in the early detection of ASD in toddlers are shown in Figure 8, Figure 9 and Figure 10. An ROC curve is a graphical representation of how the true positive rate (sensitivity) and the specificity (false positive rate) vary as the classification threshold varies. The AUC of the model is a measure of overall performance, and a higher AUC means better discriminatory performance.

The AUC of the ROC curve for the LR model is 0.9663, which indicates that the model is very good at separating the ASD and non-ASD cases. Its high sensitivity and specificity are reflected by the curve above the diagonal line (random classifier). While not as accurate as RF and SVM, the LR model still performs relatively well and could be useful when the simplicity and interpretability of the model are more important. The ROC curve of the RF model presents an outstanding AUC of 0.9994, which is very close to the ideal value of 1.0. It curves up very tightly along the top left corner, which suggests that the performance is excellent and the balance between sensitivity and specificity is near-perfect. This verifies the earlier results from the confusion matrix and precision–recall metrics, and random forest is found to be the most robust model for early ASD detection among the three. SVM’s ROC curve displays an AUC of 0.9964, which also indicates very good classification. It approaches the top left corner closely, just like RF, which indicates that the SVM model is very good at classifying between the two classes. Its high AUC complements its precision, recall, and confusion matrix performance, and it is a competitive alternative to random forest, particularly in situations requiring a high margin classification.

iii.  Implementation of Explainable AI with SHAP

To ensure transparency and interpretability in our predictive modeling, we incorporated XAI techniques using SHAP. SHAP is a framework of cooperative game theory by which each feature’s contribution to the model’s output is attributed. This also helps to understand, or at least help cover, which features are feeding into a prediction. It might be eye-opening for healthcare applications like early ASD detection; this is because, in such situations, it is important to make absolutely sure that the reasoning behind a model’s decision is trustworthy and can be clinically validated.

**Figure 11:**
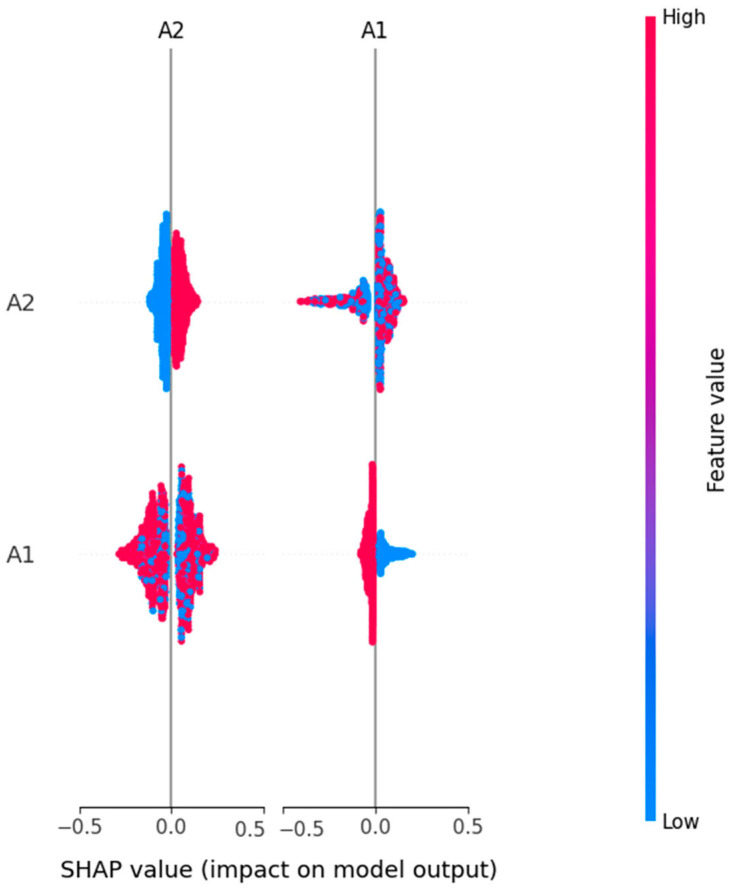
Feature impact and importance in ASD prediction using RF.

In our study, we used TreeExplainer, which is optimized for tree-based models, and used it for our random forest model, which had the best performance. Explainer_rf (X_test_scaled, check_additivity = False) was used for calculating SHAP values on the test dataset. Then, we visualized the global impact of the features via the SHAP summary plot shown in Figure 11. The effect of each feature on the model’s prediction over the entire dataset is depicted. In the summary plot, features are ordered from most to least important according to their average absolute SHAP value. Each dot represents a SHAP value, and the color represents the feature value (red for high; blue for low). This then allows us to both see which features are most influential and see their effect, with the high or low values of a feature supporting predictions for the presence of ASD (positive SHAP value) or the absence of ASD (negative SHAP value). The SHAP analysis verifies the relevance of important behavioral indicators from the dataset to give clinicians and data scientists insights into the machine learning model’s behavior. XAI integration increases the trustworthiness and efficacy of ML models in sensitive domains (e.g., pediatric healthcare and developmental screening).

**Figure 12:**
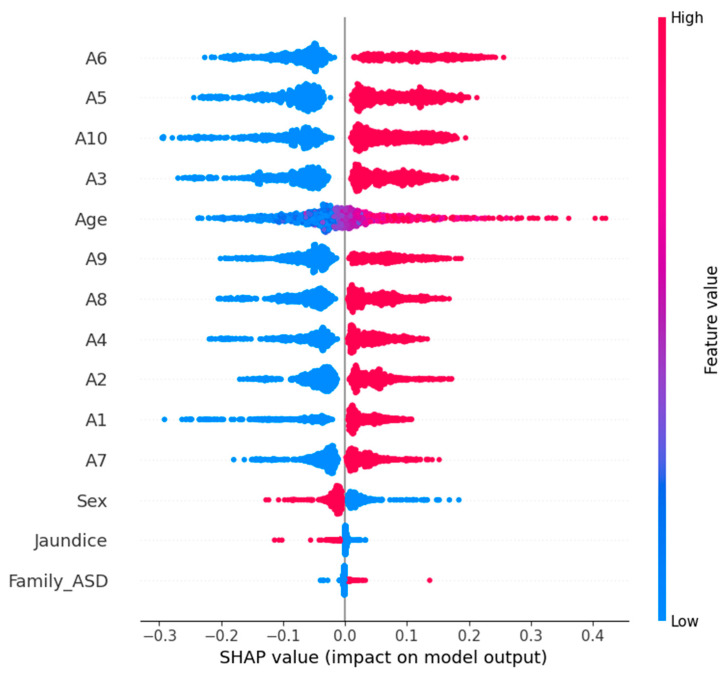
Feature impact and importance in ASD prediction using LR.

An SHAP summary plot for the logistic regression model with ASD prediction is shown in Figure 12. Global interpretation of feature contribution is provided by this visualization, such that each input feature is responsible for contributing to how the model behaves. The plot consists of a dot for each prediction, and each dot represents a SHAP value from the corresponding prediction with color encoding the initial value of the feature (red for high; blue for low). The overall importance of the features is ranked by their average absolute SHAP value.

It is also obvious from the plot which behavioral and demographic attributes had the most influence on the classification outcome of the plot. Prominently, we see features such as A5_Score, A9_Score, and A3_Score, which are likely to be very strong predictors of the logistic regression model’s decision boundary. By providing explainability in this way, transparency in machine-learning-based ASD screening can be improved; clinical experts can better understand why each prediction was made.

**Figure 13:**
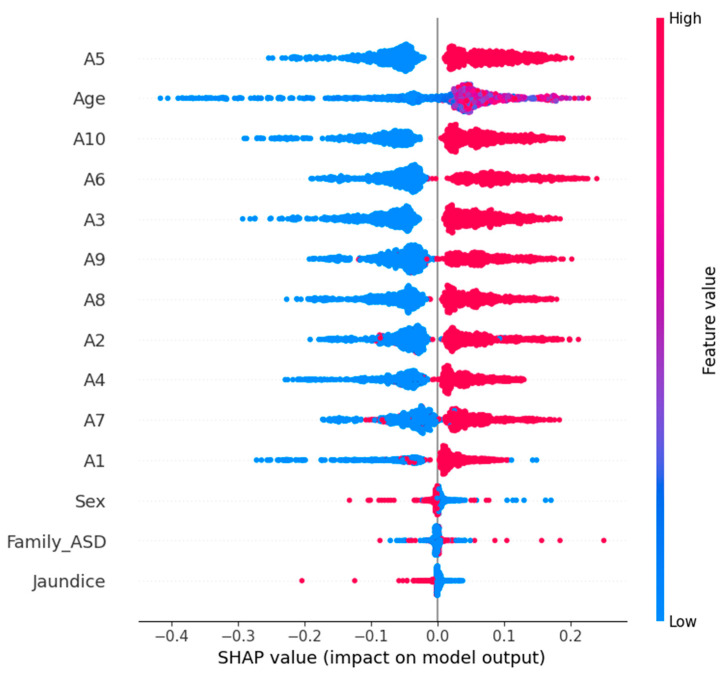
Feature impact and importance in ASD prediction using SVM.

The SHAP summary plot for the prediction of ASD is presented in Figure 13 using an SVM model. A global explainability visualization in this case enables one to see how most of the features have played a role in the model’s predictions across the test dataset. SHAP values quantify the contribution of each feature to the model’s output and are calculated using the KernelExplainer (for non-tree models, such as SVM, for example). For each point, it ranks features based on their mean absolute SHAP values, and the plot represents the whole set of predictions. The color red means feature value intensity, and blue means low feature values. In the case of points on the x-axis, the distribution of the points indicates how much of a feature pressed the prediction towards ASD (positive SHAP value) or away from ASD (negative SHAP value). A5_Score, A8_Score, and A10_Score are key features that have a great impact on the SVM’s decisions and imply their relevance in discriminating ASD cases from non-ASD cases.

In Table 3, we compare three ML models RF, LR, and SVM in terms of four main criteria: accuracy, interpretability, SHAP-driven insights and an overall verdict. The random forest model has the highest accuracy (98.85%), and this is therefore the best model based on predictive power. It has moderate interpretability, as demonstrated by the SHAP analysis, which reveals very limited insights into the decision process, only to some dominant features such as A1 and A2. On the other hand, logistic regression (accuracy: 90.53%) is highly interpretable and has a rich and diverse SHAP summary that can be very useful when we need to understand the behavior of the model. The SVM model has a balanced performance profile with high accuracy (97.70%) and good interpretability.

The use of SHAP on all the predictive models was found to be useful in increasing the model’s transparency and accountability in decision making. The LR and SVM are found to be more interpretable, and both avoid the set of features that include, among many others, A5, A6, A10, and age. The highest accuracy of 98.85% was achieved by random forest, which has no doubt relied on two features, A1 and A2, in making decisions. The most explainable model was based on performance plus transparency, namely LR. SVM also achieved a fairly good accuracy and interpretability balance, which can be very suitable for the use of explainable AI in sensitive domains such as autism screening. The three models exceeded the benchmark of acceptable predictive accuracy (above 90%) to ensure reliability in autism-screening-type classification tasks. However, the deeper analysis based on SHAP-based explainability showed the difference in model transparency and interpretability. As a result, logistic regression was found to be the most explainable model, with a clear and diverse feature importance structure that matches well to clinical and research-oriented applications. This brings transparency that is crucial in sensitive domains such as early autism detection, where knowing the decision-making process as much as the outcome itself is equally important. As a result, SVM presented a well-balanced solution with good accuracy (97.70%) and interpretability equal to logistic regression. It is therefore a good choice for deployment in the real world, where both performance and transparency are needed but not always at the cost of the other. While random forest was the most accurate (with an accuracy of 98.85%), it was much less interpretable and provided some insight through SHAP. The advantage of this model is that it works better in cases where the predictive performance is more important than the transparency of decision logic. This study confirms that the random forest model is the suitable model for early ASD detection due to its superior accuracy (98.85%) and confidence score (AUC = 0.9994). Logistic regression, although less accurate, remains valuable for interpretability-driven analysis, while SVM offers a balanced trade-off between performance and explainability.

## 5. Conclusions

This study proposed a comprehensive framework that integrates unsupervised clustering, predictive modeling, and XAI to enhance early autism detection using real-world toddler screening data. The framework systematically finds hidden patterns, accurately predicts autism, and remains transparent in its decision making, which is a must in healthcare-related domains. Initially, clustering techniques DBSCAN and K-means were used to find out the natural groupings in the data. PCA and K-means clustering provided further validation and visualization into data distribution while achieving a high silhouette score of 0.6068 with DBSCAN. Based on these clusters, we begin to integrate them into the supervised machine learning models of LR, RF, and SVM. Amongst these, the highest accuracy of 98.85% was attained using the RF model; the SVM model came second, with an accuracy of 97.70%, and the LR model had the lowest accuracy of 90.53%. Further validation of the models was confirmed by the confusion matrices and ROC curves, reporting AUCs of 0.9994 (RF), 0.9964 (SVM), and 0.9663 (LR), all of which are good. SHAP-based explainability was used to address the need for interpretability. SHAP insights from LR and SVM were clear and varied and especially suitable for the scenarios where transparency is paramount. The proposed framework is accurate and transparent, and it does a good job of discovering patterns, making it suitable for real-world autism screening scenarios. For explainability, logistic regression is suggested; SVM is suggested for balanced deployment; and random forest is suggested when maximum predictive performance is desired.

The proposed framework achieved high performance; however, there exist several limitations. The evaluation was conducted on a single dataset, which may not fully capture the diversity of real-world ASD populations. The behavioral features used were primarily questionnaire-based and lacked multimodal inputs such as speech, facial expressions, or physiological signals. The current framework relies on static data and does not account for temporal behavioral variations over time. Therefore, in the future, we aim to extend the proposed framework using longitudinal data that allows the initial detection of the trajectories of ASD over time. We also aim to implement multimodal data fusion that offers richer context for the diagnosis. By using federated learning, we can ensure the implementation of model training across multiple institutions without disseminating sensitive patient data.

## Figures and Tables

**Table 1:** Hyperparameters for each model.

Model	Hyperparameters	Description
LR	max_iter = 1000, solver = ‘lbfgs’, penalty = ‘l2’	Model convergence and regularization for linear decision boundary
RF	n_estimators = 100, max_depth = None, criterion = ‘gini’, random_state = 42	Ensemble of 100 trees for robustness and reduction in overfitting
SVM	kernel = ‘rbf’, C = 1.0, gamma = ‘scale’, probability = True	Nonlinear classification with radial basis function kernel and calibrated probabilities.

**Table 2:** Comparative analysis of accuracy, precision, recall and F1 score.

Class	Precision			Recall			F1 Score			Accuracy		
	LR	RF	SVM	LR	RF	SVM	LR	RF	SVM	LR	RF	SVM
Non-ASD (0)	0.93	0.99	0.98	0.93	0.93	0.98	0.93	0.93	0.98	0.9053	0.9885	0.9770
ASD (1)	0.84	0.99	0.96	0.85	0.98	0.96	0.84	0.98	0.96			
Macro average	0.89	0.99	0.97	0.89	0.98	0.97	0.89	0.99	0.97			
Weighted average	0.91	0.99	0.98	0.91	0.99	0.98	0.91	0.99	0.98			

**Table 3:** Comparative analysis of model accuracy, interpretability, SHAP insights, and verdicts.

Model	Accuracy	Interpretability	SHAP Insight	Verdict
RF	98.85%	Moderate	A1, A2 only	Best for performance, but less interpretable
LR	90.53%	Excellent	Diverse, clear	Best for explainability
SVM	97.70%	Good	Matches LR	Great hybrid option

## Data Availability

The original data presented in the study are openly available in a Kaggle platform with title, “Autism screening data for toddlers” [21].

## References

[1] Guo Z., Tang X., Xiao S., Yan H., Sun S., Yang Z., Huang L., Chen Z., Wang Y. (2024). Systematic review and meta-analysis: Multimodal functional and anatomical neural alterations in autism spectrum disorder. Mol. Autism.

[2] Zeidan J., Fombonne E., Scorah J., Ibrahim A., Durkin M.S., Saxena S., Yusuf A., Shih A., Elsabbagh M. (2022). Global prevalence of autism: A systematic review update. Autism Res..

[3] Zakrocka M., Gruszka M., Polańska P., Kubicka M. (2025). Autism spectrum disorder: Definition, global epidemiology, prevalence in Poland and worldwide, and heredity. J. Educ. Health Sport.

[4] Shaw K.A., Williams S., Patrick M.E., Valencia-Prado M., Durkin M.S., Howerton E.M., Ladd-Acosta C.M., Pas E.T., Bakian A.V., Bartholomew P. (2025). Prevalence and Early Identification of Autism Spectrum Disorder Among Children Aged 4 and 8 Years—Autism and Developmental Disabilities Monitoring Network, 16 Sites, United States, 2022. MMWR Surveill. Summ..

[5] Jiang Z., Li G., Zeng S., Li J., Li Y., Lin J., Fan Q. (2024). Causal Relationship between Attention-Deficit Hyperactivity Disorder and Autism Spectrum Disorder: A Two-Sample Mendelian Randomization. Br. J. Hosp. Med..

[6] Daniels A.M., Halladay A.K., Shih A., Elder L.M., Dawson G. (2014). Approaches to Enhancing the Early Detection of Autism Spectrum Disorders: A Systematic Review of the Literature. J. Am. Acad. Child Adolesc. Psychiatry.

[7] Alzakari S.A., Allinjawi A., Aldrees A., Zamzami N., Umer M., Innab N., Ashraf I. (2025). Early detection of autism spectrum disorder using explainable AI and optimized teaching strategies. J. Neurosci. Methods.

[8] Gurrapu S., Kulkarni A., Huang L., Lourentzou I., Batarseh F.A. (2023). Rationalization for explainable NLP: A survey. Front. Artif. Intell..

[9] Allam H., Makubvure L., Gyamfi B., Graham K.N., Akinwolere K. (2025). Text Classification: How Machine Learning Is Revolutionizing Text Categorization. Information.

[10] Wood N.G. (2024). Explainable AI in the military domain. Ethics Inf. Technol..

[11] Swaroop K.N., Chandu K., Gorrepotu R., Deb S. (2019). A health monitoring system for vital signs using IoT. Internet Things.

[12] Mahedy Hasan S.M., Uddin M.P., Al Mamun M., Sharif M.I., Ulhaq A., Krishnamoorthy G. (2023). A Machine Learning Framework for Early-Stage Detection of Autism Spectrum Disorders. IEEE Access.

[13] Li N., Ou J., He H., He J., Zhang L., Peng Z., Zhong J., Jiang N. (2024). Exploration of a machine learning approach for diagnosing sarcopenia among Chinese community-dwelling older adults using sEMG-based data. J. Neuroeng. Rehabil..

[14] Xiang J., Zhang J., Zheng R., Li X., Li M. (2021). NIDM: Network impulsive dynamics on multiplex biological network for disease-gene prediction. Brief. Bioinform..

[15] Ahsan M.M., Luna S.A., Siddique Z. (2022). Machine-Learning-Based Disease Diagnosis: A Comprehensive Review. Healthcare.

[16] Riad A.M., Salama A.S., Abdelaziz A., Elhoseny M. (2017). Intelligent systems based on loud computing for healthcare services: A survey. Int. J. Comput. Intell. Stud..

[17] Hartmann M., Hashmi U.S., Imran A. (2022). Edge computing in smart health care systems: Review, challenges, and research directions. Trans. Emerg. Telecommun. Technol..

[18] Ou J., Li N., He H., He J., Zhang L., Jiang N. (2024). Detecting muscle fatigue among community-dwelling senior adults with shape features of the probability density function of sEMG. J. Neuroeng. Rehabil..

[19] Zwaigenbaum L., Brian J.A., Ip A. (2019). Early detection for autism spectrum disorder in young children. Paediatr. Child Health.

[20] Rasul R.A., Saha P., Bala D., Karim S.M.R.U., Abdullah M.I., Saha B. (2024). An evaluation of machine learning approaches for early diagnosis of autism spectrum disorder. Healthc. Anal..

[21] Kaggle Autism Screening Data for Toddlers. https://www.kaggle.com/datasets/fabdelja/autism-screening-for-toddlers?select=Toddler+Autism+dataset+July+2018.csv.

[22] Deng D. (2020). DBSCAN Clustering Algorithm Based on Density. Proceedings of the 2020 7th International Forum on Electrical Engineering and Automation (IFEEA).

[23] Shah K., Patel H., Sanghvi D., Shah M. (2020). A Comparative Analysis of Logistic Regression, Random Forest and KNN Models for the Text Classification. Augment. Hum. Res..

[24] Farnaaz N., Jabbar M.A. (2016). Random Forest Modeling for Network Intrusion Detection System. Procedia Comput. Sci..

[25] Alrashdi I., Alqazzaz A., Aloufi E., Alharthi R., Zohdy M., Ming H. AD-IoT: Anomaly detection of IoT cyberattacks in smart city using machine learning. Proceedings of the 2019 IEEE 9th Annual Computing and Communication Workshop and Conference, CCWC 2019.

[26] Kaya Ş.M., İşler B., Abu-Mahfouz A.M., Rasheed J., AlShammari A. (2023). An Intelligent Anomaly Detection Approach for Accurate and Reliable Weather Forecasting at IoT Edges: A Case Study. Sensors.

[27] Taghavirashidizadeh A., Zavvar M., Moghadaspour M., Jafari M., Garoosi H., Zavvar M.H. (2022). Anomaly Detection In IoT Networks Using Hybrid Method Based On PCA-XGBoost. Proceedings of the 2022 8th Iranian Conference on Signal Processing and Intelligent Systems (ICSPIS).

